# The impact of digital transformation on resource mismatch of Chinese listed companies

**DOI:** 10.1038/s41598-024-59285-z

**Published:** 2024-04-19

**Authors:** Kedong Wu, Songzhu Liu, Mengchun Zhu, Yahui Qu

**Affiliations:** 1 School of Economics and Management, Luoyang Institute of Science and Technology, Luoyang, 471000 China; 2 Economic and Trade College, Guangxi University of Finance and Economics, Nanning, 530003 China; 3 School of Foreign Languages, Luoyang Institute of Science and Technology, Luoyang, 471000 China

**Keywords:** Digitization, Resource mismatch, Operating costs, Financing constraints, Environmental sciences, Environmental social sciences

## Abstract

Based on a microeconomic entity perspective, this paper empirically examines the effect of enterprise digitalization on resource mismatch. We found that, firstly, an increasing in enterprise digitalization reduces resource mismatch. Moreover, the results remain robust after considering endogeneity and changing the variable measurements. Secondly, enterprise digitalization can significantly reduce resource mismatch of private and large-scale enterprises and significantly contribute to reducing enterprise resource mismatch in low marketability regions and eastern regions. Thirdly, enterprise digital transformation can reduce resource mismatch by decreasing operating costs and financing constraints; Executive incentives can help reduce resource mismatch in the digital process of enterprises. Fourthly, the increase in enterprise digitalization contributes to an enhance in corporate social responsibility, and enterprise resource mismatch plays a mediating role in the relationship of enterprise digitalization development improving corporate social responsibility. Finally, in response to the findings of the study, the paper suggests countermeasures for regional and corporate countermeasures regarding digital development.

## Introduction

In the context of the fourth industrial revolution, digital technology is gradually reshaping the global economic landscape, and how to seize the opportunities of digital development and build a modern industrial system has become a focus of attention for all countries. In June 2018, Japan's Ministry of Economy, Trade and Industry released the "White Paper on Japanese Manufacturing", proposing to use digital tools to strengthen and improve the Japanese manufacturing "field force" to achieve automation, improve productivity and create new added value. The U.S. White House issued the U.S. Advanced Manufacturing Leadership Strategy in October 2018, naming the first things to be deployed in the U.S. smart manufacturing system, including smart and digital manufacturing and artificial intelligence infrastructure. China has also enacted a series of policies to promote industrial digital transformation. The 2021 Chinese government work report mentioned the need to develop the leading advantages of the digital economy and coordinate the implementation of digital industrialization and industrial digital transformation.

The implementation of industrial digital development at the enterprise level is manifested as the digital transformation of enterprises. This digital transformation represents the introduction of digital technology into the production, operation, management, R&D and innovation of enterprises, which is bound to have a strong reinvention effect on their development.

In the digital era, with the development of digital technologies such as big data, cloud computing, the Internet of Things, blockchains and artificial intelligence, digitalization has become a new engine driving economic and social development. From the perspective of economic development, digitalization exerts a positive effect on the growth^1^ and financial performance^2^ of enterprise. Additionally, digitalization plays an important role in the success of internationalization for small and medium-sized enterprises^3^. Given that digitalization is the outcome of digital technology, studies also focus on the importance of digitalization capabilities for innovation. In the process of digital transformation, enterprises have significantly increased their investments in innovation activities^4^, thus enhancing their innovation performance^5^, such as open innovation^6^and green innovation^7^. From the perspective of social development, digitalization is an effective way for enterprises to improve environmental performance^8^and ESG (environmental, social and governance) performance^9^.

Additionally, from the perspective of microeconomic entities, enterprise digitalization can weaken the power of executives, strengthen the power of grassroots efforts, and lead to the downward empowerment of corporate management, thereby promoting decentralized enterprises transformation^10^. The increase in corporate digitalization not only improves information transparency but also reduces corporate risk-taking, lowers the cost of auditing corporate financial reports^11^, and improves corporate audit quality^12^. The acceleration of the enterprise digitalization process improves the level of information sharing and the ability to absorb and integrate knowledge and therefore facilitates green technological innovation^13^, helps reduce the risk of corporate debt default^14^, and improves business performance, where the four aspects of digital transformation are corporate governance, information access, financial stability, and innovation potential to improve corporate performance^15^. In addition, digitalization has a positive impact on firms' stock liquidity, social responsibility^16^, corporate value^17^, and export quality^18^.

In summary, the current research results on enterprise digitalization are abundant, from the connotation and extension of enterprise digitalization to the measurement of enterprise digitalization and from the theoretical elaboration and model construction of enterprise digitalization to the internal impact and external effects of enterprise digitalization. The results of these viewpoints provide a solid theoretical foundation and ideas for subsequent research, but it is obvious that the current research findings have not yet covered the impact of enterprise digitalization on resource mismatch in enterprise. Modern economic theory holds that the essence of enterprise is the resource allocation mechanism. Therefore, a natural question is how will enterprises’ digital transformation affect their resource mismatch? In what ways is there heterogeneity in the effect of the enterprises’ digital transformation on their resource mismatch? What are the channels of action, regulatory paths and economic consequences of enterprise digital transformation in enterprise that affect resource mismatch? Solving the above questions can guide enterprises to develop targeted digital transformation development strategies and provide useful references for government departments to guide enterprises' digital transformation. Therefore, this paper systematically analyzes the above issues based on the existing theoretical research results and data on Chinese listed companies from 2011 to 2022, and tries to open the black box of the relationship between digitalization development of enterprise and mismatch of enterprise resource.

The possible marginal contributions of this paper lie in the following aspects. First, we provide a theoretical analytical framework and empirical evidence on the impact of enterprise digital transformation on resource mismatch, thus enriching the literature on the factors affecting resource mismatch. Second, we expand the literature on the economic consequences of enterprise digitalization by using a sample of Chinese listed firms. Third, the impact of enterprise digitalization development on resource mismatch is elucidated by analyzing the mediating role of enterprise operating costs and financing constraints.

## Theoretical mechanism and research hypothesis

### Theoretical analysis of enterprise digitalization affecting enterprise resource mismatch

Enterprises use internal and external factors of production to start production and provide products or services to the market, and technology plays an important role in this process, which is closely related to the allocation efficiency of production factors and the profit margin of enterprises^19^. Digital technologies, such as big data and Internet of Things, can improve the efficiency of factor resource utilization on the one hand and optimize resource allocation on the other hand, which is manifested in the process of the complex internal and external relationships of enterprises from imbalance to balance and from low- to high-level balance^20^. In this section, based on the understanding of digitalization as an advanced technology, the basic relationship between enterprises’ digital transformation and resource mismatch is determined.

Digital technology is an important underlying technology for technological progress in today's world, and enterprises’ digital transformation implies a high degree of integration between enterprise development and digital technology, which is manifested in the wide application and deep penetration of digital technology in product production, management, operations and after-sales service processes. These processes are often accompanied by the replacement of repetitive labor by digital technology and the replacement of traditional technology by advanced digital technology. Enterprises’ digital transformation provides a strong impetus to improve the operational efficiency of their organizations and enhances their level of automation^21^, and the enhancement of the operational efficiency and automation level of enterprises can ensure that they can carry out business activities in an orderly manner, carry out large-scale production, and at the same time broaden the boundaries of their business, thus enabling them to utilize efficiently all kinds of resources and reduce the mismatch of their resources. On the one hand, the digital development of enterprises provides convenience for the scale production of enterprises, and the scale production helps to achieve the concentration, unification and efficient use of resources. Through high-volume, organized and orderly production methods, enterprises can deploy and manage resources more effectively, reduce waste and redundancy of resources, and thus reduce enterprise resource mismatch^22^. Therefore, this paper proposes research Hypothesis 1: The digital transformation of the enterprise can reduce its resource mismatch, with other factors held constant.

### The channels of enterprise digitalization affect the enterprise resource mismatch

On the one hand, digital transformation can empower enterprises’ operations, improve their operational efficiency, and reduce resource mismatch through cost reductions. First, the application of digital technology has significantly improved the efficiency of production tools, which have entered the era of digitalization and intelligence; intelligent hardware collects data using the internet, and intelligent software digitally analyzes and makes decisions using the data according to instructions, significantly shortening machine maintenance time, downtime, and switching between processes and, thus, greatly reducing enterprises’ operations, maintenance and inventory costs. As the operating costs of enterprises are reduced, their operational efficiency will be improved, giving them a greater advantage in market competition, which will help them to better utilize and allocate their resources, and reduce the idleness and mismatch of resources^23^. Second, applying digital technologies, such as big data and artificial intelligence, enables enterprises to accurately collect and systematically analyze information from various links, including raw material procurement, product development, finished product sales, and user feedback, thus improving communications between the upstream and downstream of the industrial chain and realizing the digital management of product development and design, production and sales, significantly reducing enterprises’ production and management costs. Thus, optimal resource allocation throughout the supply chain can be realized. This also enables enterprises to deploy resources on a wider scale and make more efficient resource allocation plans, thus reducing the degree of mismatch of enterprise resources. Third, the application of digital technology can also give rise to a new mode of the sharing economy, which can not only break enterprise boundaries and integrate their production materials but also reduce the resource utilization threshold among enterprises by sharing technology, equipment and services, thus reducing the cost of cooperation among enterprises, indirectly reducing their own production costs. It also enables better utilization of resources between enterprises, reducing waste and mismatch of resources. Therefore, this paper proposes research Hypothesis 2: Other things being equal, the enterprises’ digital transformation can reduce resource mismatch by lowering costs.

On the other hand, digital transformation can reduce information asymmetry in the market, broaden corporate financing channels, alleviate financing constraints faced by enterprises, and reduce resource mismatch for enterprises. As an emerging market, China's financial system is not yet sound, and entities supplying capital often have to choose the supply object through enterprises’ information situation and financing constraints. Due to the information asymmetry between the supply and demand sides of capital, some enterprises in urgent need of financing may have difficulty obtaining or fully obtaining the funds required for their development, while some enterprises with few financial difficulties often receive excessive financing, allowing inefficient and "soft constraint" enterprises to collect funds. Such enterprises are relatively inefficient in the use of capital, and the relatively inefficient use of funds by such enterprises leads to a mismatch of their resources. Enterprises that have undergone a digital transformation have obvious advantages in information collection, transmission and utilization, which greatly improves their ability to process information. Moreover, these enterprises tend to process and export information more effectively to obtain financing and take the initiative to "push" their development status to the market, which significantly reduces the information asymmetry between the supply and demand of funds, helps enterprises broaden their financing channels, promotes competition in the banking industry, reduces the space for rent-seeking, and allows funds to reach more tail groups, thus effectively reducing enterprises’ financing constraints, improving the availability of corporate financing, enabling enterprises with urgent capital needs to obtain corresponding development funds, and reducing the possibility of high-efficiency enterprises being squeezed out of the financial market because they cannot meet financing constraints. Thus, enterprises’ overall resource mismatch is effectively reduced. Therefore, this paper proposes research Hypothesis 3: Other things being equal, digital transformation can reduce resource mismatch by alleviating enterprise financing constraints.

## Study design

### Model setting

Based on the theory above on the impact of enterprise digitization on enterprise resource mismatch, the following econometric model is set.

1 $$TFPQD_{it}^{{}} = \alpha_{0}^{{}} + \alpha_{1}^{{}} digit_{it}^{{}} + \sum\limits_{j} {\beta_{j}^{{}} } X_{ijt} + \mu_{c}^{{}} + \delta_{p}^{{}} + \nu_{t}^{{}} + \varepsilon_{it}^{{}}$$

where $TFPQD_{it}^{{}}$ represents the degree of an enterprise’s resource mismatch, and the specific calculation of this variable is explained in detail below; $digit_{it}^{{}}$ represents the firm's enterprise digitalization level; $X_{ijt}$ represents a set of control variables; $\mu_{c}^{{}}$ represents city fixed effects, $\delta_{p}^{{}}$ represents industry fixed effects, $\nu_{t}^{{}}$ represents time fixed effects, and $\varepsilon_{it}^{{}}$ represents random error terms.

2 $$Med_{it}^{{}} = \alpha_{0}^{{}} + \alpha_{1}^{{}} digit_{it}^{{}} + \sum\limits_{j} {\beta_{j}^{{}} } X_{ijt} + \mu_{c}^{{}} + \delta_{p}^{{}} + \nu_{t}^{{}} + \varepsilon_{it}^{{}}$$

3 $$TFPQD_{it}^{{}} = \alpha_{0}^{{}} + \alpha_{1}^{{}} digit_{it}^{{}} + \alpha_{2}^{{}} Med_{it}^{{}} + \sum\limits_{j} {\beta_{j}^{{}} } X_{ijt} + \mu_{c}^{{}} + \delta_{p}^{{}} + \nu_{t}^{{}} + \varepsilon_{it}^{{}}$$

where $Med_{it}^{{}}$ in Eqs. (2) and (3) denotes the mediating variable. The other variables have the same meaning as in Eq. (1).

### Variable design

#### Explained variable

Enterprise resource mismatch (*TFPQD*). In a perfectly competitive market, the marginal output of individual enterprises is equal, and their total factor productivity tends to be the same. In contrast, when firms are in an environment of market inefficiency and policy intervention, the allocation of production factors is distorted, and the misallocation of resources leads to firm productivity tending to be discrete. In this paper, the difference between enterprises’ total factor productivity and the mean value of total factor productivity at the city-year-industry level is used as a dispersion proxy variable to reflect their resource misallocation^24^. Additionally, in the robustness test, the difference between city-year-industry level total factor productivity at the 90% and 10% levels is used as a proxy variable for resource mismatch.

#### Core explanatory variable

Enterprise digitization level ($digit$). Numerous scholars have studied the measurement of the enterprise digitalization level by using indicators such as the percentage of information technology employees^25^and the percentage of intangible assets related to digitalization as a proxy for enterprise digitalization level^26^. Although the above indicators are easily accessible, it is difficult to truly measure the enterprise digitalization level. In recent years, studies on enterprise annual reports have provided ideas for the construction of the core indicators in this paper because these reports have high information content and can reflect enterprises’ development strategies^27^. Moreover, enterprises are likely to report information on their digital transformation in their annual reports^6^. Therefore, this paper has some reliability by adopting the frequency of corporate digitization-related terms in annual reports as a proxy variable for the corporate digitization level. Specifically, the text analysis method is used to construct an enterprise digital level index, and the frequency of the occurrence of digital keywords is calculated by crawling the keywords for digital transformation in the annual reports of listed enterprises through the text mining method. The digital keywords include two categories of digital underlying technology and digital application, which are shown in Table 1.

**Table 1:** Digital keyword search classification table.

Category	Keywords
Digital underlay technology	Digitization, digital technology, digital technology, big data, cloud computing, blockchain, information technology, network, Internet of Things, 5G, edge computing, automation, electronics technology, electronic technology, data integration, information technology, Internet, machine learning, computer technology, AI, 3D technology
Digital applications	digital marketing, digital currency, digital operation, digital terminal, digital trade, digital system, digital economy, digital supply chain, data information, data management, data assets, data convergence, information age, information integration, information communication, cloud service, cloud, wisdom age, wisdom construction, wisdom business, intelligence, e-commerce, cross-border e-commerce, e-commerce platform, 3D printing, 3D tools, online, online online and offline, robotics, O2O, B2B, C2C, P2P, C2B, B2C

#### Control variables

In this paper, we control for firm size, firm age, return on assets, cash flow, leverage, and the proportion of intangible assets. Among them, firm size (*size)* is expressed as the logarithm of the firm's total assets at the end of the year; firm age (*age)* is expressed as the logarithm of the firm's year of listing; return on assets (*roa)* is expressed as the ratio of the firm's total profit to total assets; cash flow (*cflow)* is expressed as the ratio of the net cash flow generated by the firm's operation to total assets; firm leverage (*finlev)* is expressed as the debt ratio of a firm; and intangible assets (*itang) is* measured as the ratio of net intangible assets to total assets of a firm.

#### Mediating variables

These variables include firm operating costs (*cost*) proxied by the firm operating cost rate; and firm financing constraints (*kz*), which draws on Kaplan and Zingales (1997): *kz* = − 1.002 * *cflow/asset* + 3.139 * *finlev* − 39. 368 *dividends/asset − 1.315 *cash/asset + 0.283 * tobin, where *cflow* represents cash flow, *asset r*epresents total firm assets, *finlev* represents firm debt ratio, *dividends* represent dividends, *cash* represents cash holdings, and tobin represents firm value.

#### Moderating variable

This variable is executive incentive (*share*), measured by the percentage of executive shareholdings.

### Data sources

In this paper, we use data on Chinese listed companies from 2011 to 2022, combined with data on the enterprise digitalization level. Among them, the data on Chinese listed companies are obtained from the CSMAR and WIND databases. The enterprise resource mismatch is calculated based on data from listed companies. The enterprise digitization level data are compiled through a web crawler method. To reduce the bias caused by extreme observations on the estimation results, a two-sided 1% shrinkage tail is applied to all continuous type variables. The descriptive statistics for each variable are shown in Table 2.

**Table 2:** Descriptive statistics of variables.

Variable	Obs	Mean	Std. dev	Min	Max
*TFPQD*	20,530	0.4376	0.3836	0.0000	2.5324
*digit*	20,530	3.2352	1.4207	0.0000	7.3376
*size*	20,530	22.0171	1.2395	19.5411	25.8301
*age*	20,529	1.9726	0.9108	0.0000	3.2581
*roa*	20,530	0.0391	0.0631	-0.2845	0.1979
*cflow*	20,530	0.0460	0.0677	-0.1598	0.2382
*finlev*	20,530	0.4002	0.2601	0.0000	0.9112
*itang*	20,530	0.0499	0.0639	0.0000	0.9242
*cost*	20,529	0.7057	0.1841	0.0011	4.0323
*kz*	16,629	0.9516	1.3431	-3.6663	4.2998
*share*	18,426	0.1621	0.2132	0.0000	0.7049

## Empirical analysis

### Baseline regression results

To estimate the impact of enterprise *digitization* on enterprise resource mismatch, this paper controls for firm-level characteristic variables while controlling for city-, industry- and year-level fixed effects in turn and reports the estimation results using progressively increasing control variables, as shown in Table 3. Column (1) of Table 3 does not add control variables as a benchmark for comparison, and it can be seen that the coefficient for *digit* is significantly negative at the 1% level, indicating that enterprises’ digital transformation reduces resource mismatch. The progressive addition of control variables in Columns (2)–(4) reveals that the *digit* coefficient is still significantly negative at the 1% level. This indicates that the increase in enterprise digitalization is indeed beneficial for reducing enterprise resource mismatch. Hypothesis 1 is verified.

**Table 3:** Baseline regression results.

	(1)	(2)	(3)	(4)	(5)	(6)	(7)	(8)
	*TFPQD*	*TFPQD*	*TFPQD*	*TFPQD*	*cost*	*TFPQD*	*kz*	*TFPQD*
*digit*	−0.0137***	−0.0121***	−0.0128***	−0.0137***	−0.0149***	−0.0116***	−0.0228**	−0.0137***
	(0.0021)	(0.0022)	(0.0022)	(0.0022)	(0.0009)	(0.0022)	(0.0098)	(0.0025)
*size*		0.0114***	0.0161***	0.0158***	0.0290***	0.0117***	0.0310***	0.0257***
		(0.0023)	(0.0024)	(0.0025)	(0.0010)	(0.0025)	(0.0112)	(0.0028)
*age*		0.0153***	0.0075**	0.0079**	0.0157***	0.0057*	0.3696***	0.0026
		(0.0033)	(0.0034)	(0.0034)	(0.0014)	(0.0034)	(0.0163)	(0.0045)
*roa*			−0.3634***	−0.3636***	−0.9191***	−0.2324***	−8.0824***	−0.2324***
			(0.0451)	(0.0459)	(0.0192)	(0.0483)	(0.2012)	(0.0572)
*cflow*			−0.3569***	−0.3352***	−0.3808***	−0.2808***	−4.2360***	−0.2925***
			(0.0414)	(0.0417)	(0.0174)	(0.0421)	(0.1809)	(0.0487)
*finlev*				0.0163	−0.0177***	0.0188	1.1472***	−0.0059
				(0.0115)	(0.0048)	(0.0114)	(0.0486)	(0.0134)
*itang*				−0.2474***	−0.1778***	−0.2220***	0.1238	−0.2643***
				(0.0422)	(0.0176)	(0.0422)	(0.2308)	(0.0462)
*cost*						0.1427***		
						(0.0168)		
*kz*								0.0095***
								(0.0029)
*_cons*	0.4819***	0.1967***	0.1393***	0.1544***	0.1534***	0.1321***	−0.0038	−0.0676
	(0.0074)	(0.0497)	(0.0497)	(0.0509)	(0.0213)	(0.0509)	(0.0817)	(0.0566)
*City*	Yes	Yes	Yes	Yes	Yes	Yes	Yes	Yes
*Industry*	Yes	Yes	Yes	Yes	Yes	Yes	Yes	Yes
*Year*	Yes	Yes	Yes	Yes	Yes	Yes	Yes	Yes
*N*	20,528	20,528	20,528	20,528	20,528	20,528	16,628	16,628
*R*^2^	0.129	0.132	0.141	0.143	0.350	0.146	0.475	0.148

Standard errors in parentheses, *p < 0.1, **p < 0.05, ***p < 0.01.

This section further tests the mechanism of the effect of enterprises’ digital transformation on resource mismatch. First, regarding verification of the role played by enterprise cost, Column (5) of Table 3 shows that the higher the degree of enterprise digitalization is, the lower is the operating cost of enterprises, and the estimated coefficient is significant at the 1% level. Column (6) of Table 3 shows that enterprises’ operating costs increase mismatches of enterprise resources, and the estimated coefficient is significant at the 1% level. Enterprises' operating costs play a partially mediating role. Second, regarding verification of the role played by corporate financing constraints, Column (7) of Table 3 shows that the higher the degree of corporate digitalization is, the lower the corporate financing constraints are, and the estimated coefficient is significant at the 5% level. Column (8) of Table 3 shows that corporate financing constraints exacerbate mismatches in corporate resources, and the estimated coefficient is significant at the 1% level. Corporate financing constraints play a partially mediating role. Hypothesis 2 and 3 are verified.

### Robustness testing

Firstly, the instrumental variables approach is used to mitigate the above problems considering the possible endogeneity bias of the model estimation caused by reverse causality and omitted variables. The mean value of firms’ digitization level in city-year-industry is re-estimated using this indicator as an instrumental variable(*digit_mean*), which satisfying the two conditions that the instrumental variable needs to satisfy. The estimation results are shown in Column (1)-(2) of Table 4. The first-stage result of two-stage least squares is reported in Column (1) of Table 4, and the result shows that the *digit_mean* coefficient of digital transformation is significant at the 1% level, demonstrating that the instrumental variable is strongly correlated with the endogenous variables. The result of the second stage regression is presented in Column (2) of Table 4, and it can be found that the *digit* coefficient in the estimation results is significantly negative at the 1% level. The above discussion shows that research Hypothesis 1 still holds after considering the endogeneity issue.

**Table 4:** Robust test I: estimation results of instrumental variable method.

	(1)	(2)
	*digit*	*TFPQD*
*digit_mean*	0.9180***	
	(0.0187)	
*digit*		−0.0171**
		(0.0067)
*size*	0.0980***	0.0162***
	(0.0075)	(0.0026)
*age*	−0.1549***	0.0073**
	(0.0101)	(0.0036)
*roa*	−0.1486	−0.3639***
	(0.1385)	(0.0459)
*cflow*	−1.1342***	−0.3394***
	(0.1255)	(0.0424)
*finlev*	−0.8162***	0.0132
	(0.0341)	(0.0129)
*itang*	−1.3046***	−0.2537***
	(0.1273)	(0.0438)
*_cons*	−1.1269***	
	(0.1617)	
*City*	Yes	Yes
*Industry*	Yes	Yes
*Year*	Yes	Yes
*N*	20,529	20,529
*R*^2^	0.430	0.018

Standard errors in parentheses, *p < 0.1, **p < 0.05, ***p < 0.01.

Secondly, to reduce the estimation bias caused by measurement errors, the measures of the main variables are changed. First, we change the measures of the core explanatory variables and adopt the digital economy development level in the cities where the listed companies are located as a proxy variable for enterprise degree of digitalization. The estimation results are presented in Column (1) of Table 5 and are consistent with those of previous papers. Second, changing the measure of the explained variables and using the difference between total factor productivity at the city-year-industry level at the 90% and 10% levels (*TFPQD1*) as a proxy for resource mismatch is estimated, and the results are presented in Column (2) of Table 5. Third, we vary the measures of both the core explanatory variables and the explained variables, and the estimation results are shown in Column (3) of Table 5. These estimation results remain consistent with those described in the previous section. The above tests show that research Hypothesis 1 still holds after considering the measurement errors of the variables.

**Table 5:** Robustness test II: Changing the measurement of variables.

	(1)	(2)	(3)
	*TFPQD*	*TFPQD1*	*TFPQD1*
*shuzijingji*	−0.0230*		−0.1008***
	(0.0127)		(0.0150)
*digit*		−0.0086***	
		(0.0026)	
*size*	0.0140***	−0.0064**	−0.0072**
	(0.0025)	(0.0029)	(0.0029)
*age*	0.0104***	0.0128***	0.0137***
	(0.0034)	(0.0040)	(0.0040)
*roa*	−0.3638***	−0.0038	−0.0011
	(0.0461)	(0.0542)	(0.0544)
*cflow*	−0.3190***	−0.0629	−0.0574
	(0.0417)	(0.0492)	(0.0493)
*finlev*	0.0290**	0.0212	0.0297**
	(0.0113)	(0.0135)	(0.0134)
*itang*	−0.2219***	−0.2969***	−0.2844***
	(0.0421)	(0.0499)	(0.0497)
*_cons*	0.3731***	1.5460***	2.5599***
	(0.1390)	(0.0601)	(0.1641)
*City*	Yes	Yes	Yes
*Industry*	Yes	Yes	Yes
*Year*	Yes	Yes	Yes
*N*	20,452	20,452	20,452
*R*^2^	0.141	0.540	0.540

Standard errors in parentheses, *p < 0.1, **p < 0.05, ***p < 0.01.

Thirdly, considering that imbalanced panel data may cause bias in the estimation results, this section has organized the imbalanced panel data into balanced panel data and re-estimated it. The results are shown in Table 6. As can be seen, the results of this article remain robust.

**Table 6:** Robustness test III: balanced panel data estimation.

	(1)	(2)	(3)	(4)
	*TFPQD*	*TFPQD*	*TFPQD*	*TFPQD*
*digit*	−0.0160***	−0.0137***	−0.0140***	−0.0159***
	(0.0026)	(0.0027)	(0.0027)	(0.0027)
*Size*		0.0229***	0.0285***	0.0299***
		(0.0028)	(0.0028)	(0.0029)
*lnage*		0.0217***	0.0189***	0.0195***
		(0.0056)	(0.0056)	(0.0056)
*Roa*			−0.3892***	−0.4079***
			(0.0551)	(0.0559)
*cflow*			−0.4246***	−0.4156***
			(0.0506)	(0.0510)
*Finlev*				−0.0123
				(0.0139)
*itang*				−0.2097***
				(0.0472)
*_cons*	0.4909***	−0.0745	−0.1588***	−0.1681***
	(0.0089)	(0.0598)	(0.0597)	(0.0611)
*N*	14,391	14,391	14,391	14,391
*R*^2^	0.151	0.157	0.168	0.170

Standard errors in parentheses, *p < 0.1, **p < 0.05, ***p < 0.01.

### Heterogeneity analysis

First, to examine the differential impact of firm digitization on firms with different property rights, we divide all firms into private and state-owned and group them for estimation. Comparing the results in Columns (1) and (2) of Table 7, we find that the *digit* coefficient is significant at the 1% level in the grouping of both private firms and state-owned firms but only contributes to the resource allocation efficiency of private firms, while the effect on the resource allocation efficiency of state-owned firms is not significant.

**Table 7:** Heterogeneity analysis I.

	(1)	(2)	(3)	(4)	(5)	(6)
	Private	State-owned	Large size	Small size	High marketability	Low marketability
*digit*	−0.0210***	0.0007	−0.0152***	−0.0077***	−0.0110***	−0.0160***
	(0.0027)	(0.0040)	(0.0033)	(0.0029)	(0.0033)	(0.0030)
*size*	−0.0018	0.0303***	0.0995***	−0.1230***	0.0145***	0.0172***
	(0.0036)	(0.0040)	(0.0042)	(0.0056)	(0.0036)	(0.0034)
*age*	0.0102**	0.0134*	0.0136**	0.0137***	0.0110**	0.0055
	(0.0042)	(0.0076)	(0.0055)	(0.0042)	(0.0050)	(0.0047)
*roa*	−0.4065***	−0.2017**	0.0552	−0.6159***	−0.4987***	−0.2767***
	(0.0530)	(0.0921)	(0.0739)	(0.0557)	(0.0714)	(0.0609)
*cflow*	−0.3512***	−0.2649***	−0.4851***	−0.2066***	−0.3703***	−0.3102***
	(0.0499)	(0.0753)	(0.0639)	(0.0521)	(0.0624)	(0.0570)
*finlev*	0.0550***	−0.0397*	−0.0650***	0.1323***	−0.0004	0.0287*
	(0.0139)	(0.0212)	(0.0173)	(0.0149)	(0.0170)	(0.0158)
*itang*	−0.2105***	−0.1385**	−0.2990***	−0.2024***	−0.2156***	−0.2771***
	(0.0662)	(0.0584)	(0.0548)	(0.0680)	(0.0618)	(0.0586)
*_cons*	0.5488***	−0.2300***	−1.7445***	3.0432***	0.1808**	0.1278*
	(0.0738)	(0.0857)	(0.0921)	(0.1146)	(0.0744)	(0.0705)
*City*	Yes	Yes	Yes	Yes	Yes	Yes
*Industry*	Yes	Yes	Yes	Yes	Yes	Yes
*Year*	Yes	Yes	Yes	Yes	Yes	Yes
*N*	13,501	7027	10,288	10,240	9401	11,127
*R*^2^	0.143	0.220	0.192	0.218	0.160	0.136

Standard errors in parentheses, *p < 0.1, **p < 0.05, ***p < 0.01.

Second, to examine the differential impact of enterprise digitization on enterprises of different sizes, we group all enterprises into large-scale and small-scale enterprises for estimation based on whether their operating income exceeds the city-, industry- and year-level median operating income of the sample. Comparing Columns (3) and (4) of Table 7, we can see that the coefficients of *digit* maintain a negative sign in the grouping of both large-scale and small-scale enterprises. From the absolute value of the coefficient, we find that the coefficient is larger in large-scale enterprises.

Third, to examine the impact of the degree of marketization on the estimation results, we use the marketization process index which is proposed by Fan Gang et al. and divide all cities into those with high and low degrees of marketization according to whether the index of the degree of marketization of the city of the enterprise exceeds the median of the degree of marketization of all cities. The estimation is done in groups. Comparing Columns (5) and (6) of Table 7, we find that the *digit* coefficient is significantly negative in the grouping of both high marketization and low marketization enterprises. From the absolute value of the coefficient, we find that the coefficient is larger in low marketability enterprises.

Fourth, to examine the differential impact of enterprise digitization on enterprises resource mismatch in different regions, we divide the enterprises into eastern, central and western region, according to the provinces where they are located. Comparing the results in Table 8, we find that the *digit* coefficient is significantly negative for both the eastern region and the middle region, but is not significant for the western region. From the absolute value of the coefficient, we find that the coefficient is larger in eastern region.

**Table 8:** Heterogeneity analysis II.

	(1)	(2)	(3)
	Eastern	Central	Western
*digit*	−0.0169***	−0.0087*	−0.0023
	(0.0027)	(0.0050)	(0.0060)
*size*	0.0129***	0.0144**	0.0245***
	(0.0031)	(0.0057)	(0.0061)
*age*	0.0117***	−0.0116	0.0076
	(0.0041)	(0.0082)	(0.0085)
*roa*	−0.3431***	−0.4001***	−0.3948***
	(0.0569)	(0.1087)	(0.1053)
*cflow*	−0.3038***	−0.3332***	−0.4499***
	(0.0513)	(0.0953)	(0.1033)
*finlev*	0.0088	0.0096	0.0467
	(0.0141)	(0.0257)	(0.0289)
*itang*	−0.2624***	−0.2146***	0.0462
	(0.0571)	(0.0767)	(0.1000)
*_cons*	0.2475***	0.1690	−0.1637
	(0.0633)	(0.1158)	(0.1247)
*City*	Yes	Yes	Yes
*Industry*	Yes	Yes	Yes
*Year*	Yes	Yes	Yes
*N*	14,336	3438	2754
*R*^2^	0.099	0.262	0.265

Standard errors in parentheses, *p < 0.1, **p < 0.05, ***p < 0.01.

### Discussion of regulatory effects and economic consequences

To consider the possible moderating effect of corporate executive incentives on the impact of corporate digitization on resource allocation efficiency, the interaction term between executive incentives and the degree of corporate digitization is added to the model, and the estimation results are shown in Column (1) of Table 9. These results show that the coefficient of the interaction term is significantly negative at the 1% level, indicating that the effect of executive incentives contributes to the effect of the corporate digitization process on resource allocation efficiency.

**Table 9:** Tests of regulatory effects and economic consequences.

	(1)	(2)	(3)
	*TFPQD*	*CSR*	*CSR*
*digit*	−0.0066**	0.0053*	0.0048
	(0.0027)	(0.0031)	(0.0031)
*share*	0.0588		
	(0.0365)		
*share#digit*	−0.0322***		
	(0.0094)		
*TFPQD*			−0.0366***
			(0.0101)
*size*	0.0234***	0.1372***	0.1379***
	(0.0027)	(0.0035)	(0.0036)
*age*	−0.0100**	−0.0272***	−0.0269***
	(0.0040)	(0.0048)	(0.0048)
*roa*	−0.3039***	7.3418***	7.3282***
	(0.0518)	(0.0823)	(0.0824)
*cflow*	−0.2795***	−0.0762	−0.0878
	(0.0443)	(0.0610)	(0.0611)
*finlev*	0.0398***	−0.0269	−0.0262
	(0.0120)	(0.0165)	(0.0165)
*itang*	−0.2587***	0.1857***	0.1753***
	(0.0464)	(0.0605)	(0.0606)
*_cons*	−0.0123	−0.2894***	−0.2860***
	(0.0564)	(0.0729)	(0.0729)
*City*	Yes	Yes	Yes
*Industry*	Yes	Yes	Yes
*Year*	Yes	Yes	Yes
*N*	18,426	19,757	19,757
*R*^2^	0.145	0.445	0.445

Standard errors in parentheses, *p < 0.1, **p < 0.05, ***p < 0.01.

In addition, with the development of society and the progress of the times, enterprises not only pursue their own profits, but also corporate social responsibility has become an important dimension to measure enterprises. Therefore, this section further studies the economic consequences of reducing resource mismatch from the perspective of corporate social responsibility. Thus, we verify that corporate digitization helps improve corporate social responsibility (*CSR*), and the estimated results are presented in Column (2) of Table 9. The coefficient of corporate digitization is found to be significant at the 10% level, which is consistent with existing studies. Combined with the findings of this paper, enterprise digitization can reduce the resource mismatch. A natural idea is whether the reduction of enterprise resource mismatch contributes to an increase in corporate social responsibility. For this purpose, the degree of enterprise digitization and enterprise resource allocation efficiency indicators are brought into the model simultaneously, as shown in Column (3) of Table 9. The results show that the decrease in enterprise resource mismatch contributes to an increase in the value of the corporate social responsibility indicators, thus indicating that enterprise resource mismatch in the relationship of enterprise digital development improving *CSR* plays a mediating role, indirectly indicating the reasonableness of the findings of this paper.

### Summary and discussion of empirical analysis

The empirical analysis shows that the increase in enterprise digitalization is indeed beneficial for reducing enterprise resource mismatch. From the estimation of control variables in Column (1) of Table 3, the higher the return on assets is, the more abundant the cash flow is, and the higher the proportion of intangible assets is, the lower the resource mismatch of enterprises is. This means that improving the return on assets, cash flow, and proportion of intangible assets can help reduce resource mismatch in enterprises, and these indicators need to be given sufficient attention by enterprises. The estimated coefficient of firm size and age have a negative effect on the firm’s resource mismatch, indicating that to a certain extent, there is a greater mismatch of the resource mismatch between "larger and older enterprises" than that of other enterprises. This implies that large-scale enterprises and those established for a long time should pay more attention to the issue of mismatched enterprise resources.

The operating costs and financing constraints of enterprises play a mediating role, with the mediating effect accounting for 15.52% and 1.58% of the total effect, respectively. This suggests that reducing operational costs and alleviating financing constraints through digital transformation is an important means of reducing resource mismatch in enterprises. What’s more, the role of executive incentives contributes to the impact of the corporate digitization process on resource allocation efficiency. Thus, In the process of digital transformation, enterprises should pay attention to motivating executives and maximize their important role in reducing resource mismatch.

The decrease in enterprise resource mismatch contributes to an increase in the value of the corporate social responsibility indicators, thus implying that enterprise resource mismatch in the relationship of enterprise digital development improving *CSR* plays a mediating role. It means that reducing the mismatch of enterprise resources is not only beneficial for the development of the enterprise itself, but also helps to strengthen its social responsibility. This further indicates that this study has strong practical value.

## Research conclusions and policy recommendations

### Research findings

In the context of China's economy shifting from high growth to high-quality development, how to effectively reduce enterprise resource mismatch is a topic that needs to be studied urgently. This paper first compares the theoretical mechanism that enterprise digitization affects enterprise resource mismatch and proposes research hypotheses. Second, the empirical analysis is conducted based on data on Chinese listed companies from 2011 to 2022, which found that, first, the improvement in enterprise digitization contributes to the reduction in enterprise resource mismatch, and the results remain robust after considering endogeneity issues, changing the measurement methods of variables and other treatments. Second, heterogeneity studies show that enterprise digitization can significantly reduce the resource mismatch of private, large-scale enterprises while significantly contributing to reduction in enterprise resource mismatch in low marketability regions and eastern regions. Third, enterprise digital transformation can reduce enterprise resource mismatch by decreasing enterprises’ operating costs and financing constraints. Fourth, executive incentives contribute to the impact of the enterprise digitalization process to reduce the resource mismatch. Fifth, the increase in enterprise digitalization contributes to the improvement of corporate social responsibility, and enterprise resource mismatch plays a mediating role in the role of enterprise digital development in improving corporate social responsibility.

### Policy recommendations

First, the government should actively promote the reform of institutional mechanisms to provide a favorable external environment for the digital transformation of enterprises. The introduction of targeted financial and taxation policies to help labor-intensive enterprises complete intelligent and digital transformations and actively build a big data sharing platform to achieve a seamless connection between enterprise resources and the internet platform to share production materials, information and consultations reduces the difficulty and cost of enterprises’ digital transformations, thus reducing the resource mismatch. At the same time, we improve the legal and regulatory system in the field of digitalization, accelerate the development of the systems related to data resources, and vigorously protect the intellectual property rights of digital technology to stimulate enterprises’ potential to reduce the resource mismatch.

Second, enterprises should play a role in digital development to reduce the resource mismatch. The innovation and application of digital technology should be accelerated, and the potential of digital technology in enterprise transformations and to upgrade and reduce the resource mismatch should be continuously released. At the same time, enterprises should accelerate the construction of digital intelligent factories, strengthen the layout of the digital infrastructure, increase investments in R&D, introduce talent in the field of digital technology, and create an efficient digital sharing platform to realize the efficient transmission and use of information, ultimately improving the integration ability of enterprises for production factors, reducing the operating costs and financing constraints of enterprises, and helping enterprises reduce the resource mismatch. The company's digital sharing platform ultimately improves the integration ability of production factors, reduces operational costs and financing constraints, and helps enterprises reduce the resource mismatch.

Third, central and western regions and noncoastal provinces should pay attention to the digital development of enterprises in their areas, actively learn from the developed eastern regions, and take advantage of the opportunity presented by digital development to continuously reduce enterprise resource mismatch. At the same time, they need to make scientifically plan for the key direction of their own development, realize their own digital upgrade to compete, and empower enterprises to reduce their operating costs and financing constraints.

## Research shortcomings and future perspectives

On the one hand, this article lacks comprehensive research on the mechanism of reducing enterprise resource mismatch through digital transformation. There may be many mechanisms for digital transformation to reduce the mismatch of enterprise resources, and this article will only study two of them. This could be one of the future research directions.

On the other hand, this paper is not specific enough to study the resource mismatch of enterprises. In the future, further research can be conducted on the mismatch of labor and capital in enterprises.

## Data Availability

The data supporting the findings of the present study are available from the corresponding author upon reasonable request.
